# Meal kit subscription services and opportunities to improve family vegetable consumption

**DOI:** 10.1093/heapro/daad155

**Published:** 2023-11-22

**Authors:** Kylie Fraser, Penelope Love, Rachel Laws, Karen J Campbell, Alison Spence

**Affiliations:** Faculty of Health, School of Exercise & Nutrition Sciences, Deakin University, Melbourne Burwood Campus, 221 Burwood Highway, Burwood, Victoria 3125, Australia; Faculty of Health, School of Exercise & Nutrition Sciences, Deakin University, Melbourne Burwood Campus, 221 Burwood Highway, Burwood, Victoria 3125, Australia; Faculty of Health, Institute for Physical Activity and Nutrition (IPAN), Deakin University, Melbourne Burwood Campus, 221 Burwood Highway, Burwood, Victoria 3125, Australia; Faculty of Health, School of Exercise & Nutrition Sciences, Deakin University, Melbourne Burwood Campus, 221 Burwood Highway, Burwood, Victoria 3125, Australia; Faculty of Health, Institute for Physical Activity and Nutrition (IPAN), Deakin University, Melbourne Burwood Campus, 221 Burwood Highway, Burwood, Victoria 3125, Australia; Faculty of Health, School of Exercise & Nutrition Sciences, Deakin University, Melbourne Burwood Campus, 221 Burwood Highway, Burwood, Victoria 3125, Australia; Faculty of Health, Institute for Physical Activity and Nutrition (IPAN), Deakin University, Melbourne Burwood Campus, 221 Burwood Highway, Burwood, Victoria 3125, Australia; Faculty of Health, School of Exercise & Nutrition Sciences, Deakin University, Melbourne Burwood Campus, 221 Burwood Highway, Burwood, Victoria 3125, Australia; Faculty of Health, Institute for Physical Activity and Nutrition (IPAN), Deakin University, Melbourne Burwood Campus, 221 Burwood Highway, Burwood, Victoria 3125, Australia

**Keywords:** meal kits, family nutrition, family meals, vegetable intakes, home cooking

## Abstract

Popular commercial meal kit subscription services (MKSSs) may support families to overcome barriers to cooking and eating at home, and facilitate improved vegetable consumption. The global meal kit market has expanded rapidly creating a gap in our understanding of the health-promoting potential of MKSSs. This paper describes the contemporary MKSS market in Australia and provides a vegetable-specific content analysis of a sample of recipes. A 1-week subscription was purchased for all Australian-based MKSSs (*n* = 9) and websites were systematically reviewed to document key characteristics and recipe features. Vegetable content of all available recipes within a 1-week period were analysed. Our findings highlight the rapid expansion and evolution of MKSS market offerings over the past decade and their potential to support family vegetable consumption. Across all analysed recipes (*n* = 179), MKSSs provided a median of 2.5 vegetable serves per person (range 0.7–7.5 serves) and a median of 3 different types of vegetables from 2 vegetable subgroups (i.e. dark green, red and orange, starchy, legumes and all other vegetables). This suggests that MKSSs may support family vegetable consumption if families select recipes with a greater number and variety of vegetables. However, an opportunity remains for MKSSs to improve both vegetable quantity and variety to positively influence population health. Further research is required to describe how families use meal kits promoting a greater understanding of their potential to improve family nutrition.

**Contribution to Health Promotion**

- Provides new insights regarding the Australian meal kit market including growth and diversity of offerings across a wide range of price points.
- Quantifies vegetable serve numbers and variety across nine meal kit companies providing insights into opportunities to meet daily vegetable recommendations.
- Examines opportunities to leverage this knowledge to support users to maximize vegetable consumption.

## BACKGROUND

Globally, people consume insufficient quantities of vegetables, fruit and wholegrains (Micha *et al.*, 2015). The benefits of diets rich in vegetables are well established (Wang *et al.*, 2014). Greater intakes of vegetables are associated with a reduced risk of obesity and non-communicable diseases such as cardiovascular disease and some cancers (Aune *et al.*, 2017). Vegetable-rich diets are also more environmentally sustainable (Nelson *et al.*, 2016). Accordingly, the World Health Organisation (WHO, 1990) and dietary guidelines worldwide recommend vegetable consumption higher than current population intakes (Cámara *et al.*, 2021; FAO, 2023).

The Australian Dietary Guidelines (ADGs) recommend children consume 2.5–4.5 serves of vegetables (188–338 g, dependent on age) per day, increasing to 5 or more serves in adulthood, to promote optimal nutrition (NHMRC., 2013). The ADGs also recommend the consumption of a wide variety of vegetable types and colours to optimize the diversity of nutrients consumed (NHMRC, 2013). However, similar to global vegetable intakes for many Oceanic, American and European countries (~186 g/day) (Kalmpourtzidou *et al.*, 2020), Australians of all ages consume substantially fewer vegetables than recommended (ABS, 2012). On average, Australian adults consume 3.0 serves of vegetables per day and children (2–18 years) consume <2.0 serves per day (ABS, 2017).

The family meal environment presents an opportunity to influence vegetable intakes of adults and children. In many countries including America (Moore *et al.*, 2016), Norway (Vejrup *et al.*, 2008) and Australia (Rebuli *et al.*, 2020), vegetables are mostly consumed at the evening meal (dinner). Frequent consumption of home-cooked meals has been associated with healthier dietary intakes, including greater vegetable (Mills *et al.*, 2017; Wolfson *et al.*, 2020) and less fast food (Wolfson and Bleich, 2015) consumption. Cooking at home has been identified as a key strategy to promote healthy dietary intakes among families (Condrasky *et al.*, 2006; Wolfson and Bleich, 2015; Mills *et al.*, 2018). Furthermore, consuming home-cooked family meals provides opportunities for parents to influence children’s lifelong eating behaviours and food preferences (Birch and Fisher, 1998). For example, family meals provide an opportunity for parental modelling (Dallacker *et al.*, 2019), repeated exposure (Wolfenden *et al.*, 2021) and children’s involvement in meal preparation (Dallacker *et al*., 2019), all of which are positively associated with greater vegetable consumption.

Parents frequently report barriers to cooking and eating at home such as a lack of time, energy and skills/knowledge (Fulkerson *et al.*, 2008, 2011; Beshara *et al.*, 2010; Dwyer *et al.*, 2015; Lavelle *et al.*, 2016; Middleton *et al.*, 2020; Oberle *et al.*, 2020). Global trends suggest families are cooking less and relying on convenience meals (i.e. frozen/microwave meals) and fast foods in the provisioning of family meals (Banwell *et al.*, 2005; Smith *et al.*, 2013; Baker *et al.*, 2020; Cameron *et al.*, 2022). These foods generally contain fewer vegetables than home-cooked meals (Lachat *et al.*, 2012) and are linked to increased weight gain (Nago *et al.*, 2014) and poor diet quality (Golper *et al.*, 2021). Given the potential for home-cooked meals to support family vegetable consumption and improved diet quality, while also providing some time efficiencies, an exploration of meal kit delivery services that may reduce barriers and enable cooking at home is warranted.

Meal kit subscription services (MKSSs) are increasingly popular, expanding rapidly across the globe and Australia in the past decade (Lee and Ham, 2021; Line, 2021; Robinson-Oghogho *et al.*, 2022). During the coronavirus pandemic, MKSSs globally reported substantial increases in orders (Lee and Ham, 2021) including prominent Australian MKSSs HelloFresh™ and Marley Spoon™ (B&T Magazine, 2020; Mitchell, 2020). MKSSs provide convenience and time-savings in the provisioning of home-cooked meals through online meal planning, selection and delivery of predominately fresh/pre-portioned ingredients paired with recipes. They offer subscribers flexibility in the number of meals (i.e. two to six meals per week) and serving sizes (i.e. one to six serves per meal) based on their household’s needs.

Emerging research suggests that MKSSs may influence food provisioning behaviours (e.g. home cooked meals) by reducing common barriers to family meal provisioning (Utter and Denny, 2016; Utter *et al.*, 2019; Moores *et al.*, 2020; Fraser *et al.*, 2021; Robinson-Oghogho *et al*., 2022). A recent qualitative study of primary meal providers (*n* = 16) from Australian families reported that MKSSs decreased food-related decision-making fatigue and mental load (Fraser *et al*., 2021). Some participants reported reduced consumption of convenience/fast foods and increased family vegetable consumption (Fraser *et al*., 2021). MKSSs also created opportunities for the involvement of children (2–18 years) and/or partners in meal planning and preparation tasks (Fraser *et al*., 2021). Two pilot interventions, both from New Zealand (Utter and Denny, 2016; Utter *et al*., 2019), reported similar findings indicating that the provision of MKSSs (i.e. My Food Bag™ and Bargain Box™) facilitated involvement of adolescents in family meal preparation. Cross-sectional and experimental studies among primary school-aged children provide supporting evidence for the associations between involvement in family meal preparation and increased vegetable preferences (Chu *et al.*, 2013, 2014; Asigbee *et al.*, 2020), exposure (Asigbee *et al*., 2020) and intakes (Chu *et al*., 2013, 2014; Asigbee *et al*., 2020). MKSSs may therefore influence children’s vegetable consumption through several learning pathways such as modelling, exposure and hands-on experience.

MKSSs are strongly marketed as healthy alternatives to ready-to-eat food purchased away from home (e.g. convenience meals and fast food). Qualitative interviews with families from Denmark (*n* = 13) (Hertz and Halkier, 2017) to Australia (*n* = 16) (Fraser *et al*., 2021) indicate MKSSs are generally perceived to be healthy and nutritious because they include fresh ingredients and vegetables. Two recent nutritional analyses of several Australian MKSSs suggest the meals on average provide 2.3–3.1 vegetable serves per person (Gibson and Partridge, 2019) and include a median of three vegetables per meal (Moores *et al*., 2020). One study reviewed 60 recipes (3 meals/week for 4 weeks) across five MKSS providers (Gibson and Partridge, 2019), while the other analysed 251 recipes from a single provider (HelloFresh™) over a 12-month period (Moores *et al*., 2020).

These findings suggest that MKSSs may have the potential to positively influence family vegetable intakes. However, a gap exists in our current knowledge of the Australian market due to the dynamic nature of MKSSs (38–40). A greater understanding of the contemporary market, including products and services, is required to gain a complete picture of their potential to be harnessed as a health-promoting tool. Furthermore, given MKSSs offer many opportunities known to influence vegetable consumption, an exploration of the vegetable quantity and variety of all Australian MKSSs provides insights into the market’s potential to improve family vegetable consumption. Therefore, the aim of this study is to provide a comprehensive overview of MKSSs in Australia and to assess the vegetable content of a sample of recipes.

## METHODS

### MKSS sample and inclusion criteria

A structured search using Google Chrome and Boolean logic was conducted in March 2022 to identify MKSSs operating in Australia. Search terms included a combination of: ‘meal kits’, ‘food boxes’, ‘recipe box’, ‘delivery’ and ‘subscription services’. MKSSs were defined as a subscription service that delivers predominately fresh, pre-measured ingredients paired with recipes and directly to consumers to prepare at home. Services that did not involve home meal preparation and cooking (i.e. pre-prepared or ready-to-heat meals) were excluded.

A 1-week subscription which included the delivery of a ‘meal kit’ (i.e. ingredients and recipes to prepare meals at home) was purchased between May and June 2022 from each provider enabling access to website content and recipes that are only available with a paid subscription. For pragmatic reasons, the number of meals and servings ordered by the primary author (K.F) was based on the minimum serving requirements across all MKSSs (i.e. three meals for two people). Purchasing of meals provided the opportunity to access all recipes within each MKSS from which we sampled 1 week of recipes (i.e. all recipes available for the company in 1 week). The recipes received in the meal kit deliveries were not the subject of data analysis, therefore the meals delivered were chosen based on personal preference. Where the MKSS did not operate in the primary author’s (K.F) hometown (*n* = 4), a co-author or research assistant was the recipient. Ethics approval was not required for this study as data were publicly available and did not include human participants.

### Data collection

#### MKSS characteristics and features

MKSS websites were systematically reviewed between May and June 2022, and data extracted into a Microsoft Excel spreadsheet. Data extracted included company name, year company launched in Australia, geographical locations serviced, number of people and meals available to order, number of weekly meal options, cost per week (as of May/June 2022 for a meal kit to feed two people for three meals), meal types (i.e. vegetarian) and extra services (e.g. marketplace to purchase additional foods and meal customization incurring additional costs).

For each MKSS, all recipes available in a 1-week period between April and May 2022 were downloaded from MKSS websites as a sample for that provider. Data extracted from recipes included key features such as inclusion of nutritional information, number of ingredients and cooking steps, visual presentation (i.e. ingredients and/or cooking steps) and preparation time.

#### Vegetable variety (type and subgroups) of recipes

The vegetable variety of each downloaded recipe was extracted into a specifically designed Microsoft Excel spreadsheet. Data were recorded for the number of different types of vegetables within the ingredient list and the number of different vegetable subgroups represented. For example, if a recipe included one carrot and half a pumpkin, this was recorded as two different types of vegetables but one vegetable subgroup (i.e. red- and orange vegetables). The definition of ‘vegetables’ used follows the ADGs, which includes all fresh unprocessed (e.g. leafy green vegetables, cruciferous/brassica vegetables, starchy root and tuber vegetables, gourd vegetables, tomato, capsicum and allium vegetables) or minimally processed vegetables (including canned legumes/beans), with the exception of tofu which is classified as a ‘meat alternative’ (NHMRC, 2013). The five vegetable subgroups were classified as dark green vegetables, red and orange vegetables, starchy vegetables, legumes and all other vegetables.

#### Meal kit delivery

Data documented from meal kit deliveries was used in the analysis only for cross-checking purposes (e.g. checking Foodworks vegetable weights). A standardized data collection protocol was used to extract data and document the recipes and ingredients received in the ‘meal kit’ deliveries. This included taking photographs of recipe cards and ingredients provided for each MKSS delivery. Vegetables were weighed using kitchen scales to the nearest gram.

#### Recipe data entry and vegetable quantity data collection

The weights of all ingredients for each downloaded recipe were entered by the primary author (K.F) into an Australian nutritional analysis software program, Xyris FoodWorks (FoodWorks, 2019). Foodworks combines several comprehensive Australian nutritional composition databases (AUSNUT2013, AusFoods 2019 and AusBrands 2019). The software was deemed suitable as the nutritional databases are appropriate to the Australian region and this program has been used previously for recipe analysis (Croxford, 2017; Dickinson *et al.*, 2018; Wademan *et al.*, 2020; Jones *et al.*, 2021), including MKSS recipes (Gibson and Partridge, 2019).

A standardized protocol and coding rule hierarchy were developed, informed by other studies (Croxford, 2017; Dickinson *et al*., 2018; Gibson and Partridge, 2019; Wademan *et al.*, 2020; Jones *et al*., 2021), photographs and weights of ingredients from sample meal kit deliveries, and regular review by the research team. Only coding and analysis of vegetables is relevant to this paper and described here.

If an ingredient was unavailable in FoodWorks, a nutritionally equivalent ingredient was substituted (e.g. Pak choy was substituted with bok choy/choy sum variants). Product nutrition information panels were utilized to calculate drained weight of canned/jarred vegetables (e.g. chickpeas). Additional information was obtained from supermarket websites as required (i.e. number of ‘spring onions’ in one bunch). Vegetable ingredients/foods classified as ‘discretionary’ by the ADGs (NHMRC., 2013) and national nutrition survey (ABS, 2013) (e.g. vegetable-based dips, pickles, relishes and savoury sauces) were excluded from analysis.

Where an ingredient weight was provided in the recipe, that was the weight entered in Foodworks. More commonly, vegetables were referred to in recipes by unit description (e.g. ‘1 carrot’ or ‘2 potatoes’). To standardize data entry, the FoodWorks ‘medium’ portion size was applied. Cross-checking of Foodworks medium weights with meal kit delivery samples identified three instances where Foodworks ‘medium’ size was >50% larger than the average recorded weight of vegetables received during meal kit deliveries, and in these instances the latter weight was utilized. This was applied to sweet potato (269 g per potato was entered), capsicum (130.5 g edible portion per capsicum was entered) and leek (99 g per leek was entered).

For vegetables with quantities not specified and unavailable in Foodworks (e.g. ‘1 small bag of mixed leaves’ or ‘1 head broccoli’), the quantity applied during data entry was sourced from the average weight recorded during meal kit deliveries. For example, the weight of ‘cos lettuce’ was not specified on MKSS recipes (*n* = 7) and was not available in Foodworks, therefore the average weight of three cos lettuces (223 g) received during meal kit delivery was applied in these instances. For vegetables on downloaded recipes that were not received during meal kit delivery (i.e. could not be weighed), the quantity was sourced from other MKSS recipes and the average was applied. For example, the weight of cauliflower reported on five recipes was averaged (300 g) and applied to two recipes with unspecified cauliflower quantity.

All coding was conducted by the first author (K.F). A second author (A.S), a dietitian with prior experience using FoodWorks, conducted a cross-check of 39 recipes (20%) at both extremes of the distribution, that is <1.0 vegetable serve per person (*n* = 14) and >5.0 vegetable serves per person (*n* = 25). Any discrepancies in FoodWorks outputs (i.e. vegetable serve calculations) or uncertainties in coding were discussed and resolved by the coders (K.F and A.S).

### Data analysis of vegetable content

Data pertaining to the vegetable content of each recipe was extracted from FoodWorks to enable a per person analysis using Stata version 17 (StataCorp, 2019). Due to the skewed distribution of the data, the descriptive statistics reported are median, minimum, maximum, inter-quartile range and percentages. Data analysis included number of vegetable serves per person and vegetable variety, defined as the number and type of vegetables (i.e. vegetable subgroups) provided per recipe. Garlic, fresh herbs, spring onion and tomato paste were included in vegetable serve calculations but excluded from vegetable variety analyses, as their small quantities but frequent use may have artificially raised vegetable variety scores.

Continuous values for vegetable serves and vegetable subgroup serves were automatically classified and calculated by FoodWorks software (FoodWorks, 2019). The vegetable serves calculated by FoodWorks were informed by the ADGs modelling system which translates Nutrient Reference Values (NRV) into estimated food group requirements for optimal health and well-being (NHMRC, 2016). The dietary modelling system derives serving sizes of vegetable subgroups based on their relative energy density. As such, FoodWorks differentiates vegetables into five subgroups: dark green vegetables (100 kJ/serve), red and orange vegetables (150 kJ/serve), starchy vegetables (250 kJ/serve), legumes (350 kJ/serve) and other vegetables (100 kJ/serve). For example, eating 250 kJ of broccoli would equate to 2.5 serves of vegetables per person compared with eating 250 kJ of potato which would equate to 1.0 serve of vegetables per person, as ‘dark green’ vegetables are less energy dense than starchy vegetables. Utilizing energy rather than weight values of vegetables to determine serves was aligned to the entry of raw weights from the recipes. One manual adjustment was made, as FoodWorks classifies sweet potato as a ‘red and orange’ vegetable, but the ADGs classifies it as ‘starchy’. Given the relatively large quantities of sweet potato in many of the high-vegetable content recipes, and the potential overestimation, vegetable serves for all instances of sweet potato were manually calculated using 250 kJ/serve and imputed.

Median vegetable serves across the meals provided per MKSS were also assessed as a categorical variable represented by ‘≤1.0 serve’, ‘1.1 to ≤2.0 serves’, ‘2.1 to ≤3.0 serves’, ‘3.1 to ≤4.0 serves’ and ‘≥4.1 serves’. A vegetable subgroup variety score between 0 and 5 was also calculated for each recipe and reported per MKSS, with one point awarded for each vegetable subgroup.

## RESULTS

### MKSS characteristics and features

Table 1 provides an overview of the nine MKSSs identified, and their main characteristics and recipe features. Four MKSSs were owned by multi-national corporations and operated Australia-wide, and five were Australian-owned. Two of the multi-national MKSSs (Dinnerly™ and EveryPlate™) were introduced to the Australian market as lower-price point options to complement higher-priced sister companies (Marley Spoon™ and HelloFresh™). Most MKSSs offered meal plans to feed two to four people for three to five nights per week. During the study period, the MKSSs offered 12 (You Plate It™) to 50 (Dinnerly™) weekly recipes. The total cost per week for a meal kit to feed two people for three nights ranged from AUD$48.93 to AUD$89.00, equating to a cost of AUD$8.12 (EveryPlate™) to AUD$14.83 (You Plate It™) per person per meal.

**Table 1:** Meal kit subscription service (MKSS) characteristics and recipe features (recorded in May/June 2022)

	Dinnerly™	Marley Spoon™	HelloFresh™	EveryPlate™	Dinner Twist™	You Plate It™	My Foodie Box™	Pepper Leaf™	Dinner Sorted™
Meal kit characteristics (including ordering options)									
Geographical locations Serviced^a^	Nation-wide	Nation-wide	Nation-wide except TAS	Nation-wide except NT, SA, TAS	WA (Perth)	WA (Perth)	WA (Perth)	NSW, QLD and VIC	VIC (Melbourne)
Launched in Australia	2018	2015	2012	2020	2012	2014	2017	2015	2014
Number of people	2 or 4	2 or 4	2 or 4	2, 4 or 6	2 or 4	2, 3 or 4	2 or 4	1, 2, 3, 4 or 6	2 or 4
Number of meals per week	2, 3, 4, 5 or 6	2, 3, 4 or 5	3, 4 or 5	3, 4, 5 or 6	3, 4 or 5	2, 3, 4 or 5	3, 4 or 5	2, 3, 4 or 5	3, 4 or 5
Number of recipes per week^b^	50	45	34	22	16	12	19	16	17
Cost (AUD) per week^c^	53.99	79.49	75.93	48.93	82.50	89.00	79.95	77.90	79.00
Cost (AUD) per person/meal^d^	8.99	13.24	12.66	8.12	13.75	14.83	13.33	12.80	13.16
Specific ‘Meal Types’ available to order^e^	Vegetarian and vegan, family friendly and kid-approved, low-calorie, balanced, reduced-carb, feed-a-crowd	Vegetarian and vegan, family friendly, low-calorie, balanced, reduced-carb	Vegetarian, family friendly, calorie smart, carb smart, premium, gourmet^f^	Family, vegetarian, premium, slow cook	Fast and family friendly, meat-free, nourishing and delicious, favourites	Vegetarian, kid-friendly	Vegetarian and vegan, family favourite,	Vegetarian, almost ready meals	Veggie, family, low-calorie
Extra services/meals that incur additional costs^g^	Marketplace to purchase grocery items, ready-to-heat meals, slow cooker meals, desserts, snacks and seasonal specials (i.e. brunch, lunch options).	Fruit box, Marketplace to purchase grocery items, ready-to-heat meals, desserts, snacks and seasonal specials (i.e. brunch, lunch options).	Fruit box, ready-to-heat meals, soups gourmet/premium meals, sides, desserts, breakfast and brunch specials	Fruit box	Marketplace to purchase grocery items such as foods for breakfast, snacks, bakery foods, desserts, ready-to-heat meals and soups, and fruit and vegetable boxes	Marketplace to purchase grocery item, ready-to-heat meals	None	None	Juice kits, Fruit, Drinks, Desserts
Recipe features									
Type of nutrition information provided	Per serving: energy (kcal), fat, carbohydrates and protein	Per serving: energy (kcal), total fat, carbohydrates and protein	Per serving/100 g: energy (kJ/Cal), protein, total fat, saturated fat, carbohydrates, sugars and sodium	Per serving/100 g: energy (kJ), protein, total fat, saturated fat, carbohydrates, sugars and sodium	Per serving: protein, total fat and carbohydrates	Per serving: energy (Cal)	Per serving/100 g: energy (kJ/kcal), protein, total fat, saturated fat, carbohydrates, sugars and sodium	None	None
Number of prep/cooking steps (range)	5	6	4–6	4	4–6	5–6	5–6	4–6	6–7
Visual presentation (i.e. meal, ingredients and/or cooking steps)	Picture of meal	Pictures of meal and cooking steps	Pictures of meal, ingredients and cooking steps	Pictures of meal, ingredients and cooking steps	Pictures of meal and cooking steps	Pictures of meal, ingredients and cooking steps	Pictures of meal and cooking steps	Picture of meal	Picture of meal
Number of ingredients provided per recipe (median, range)	10 (6–13)	12 (9–16)	14 (9–17)	12 (9–16)	10 (9–13)	12 (6–14)	13^f^ (8–14)	7^f^ (6–11)	9^f^ (7–12)
Reported time to prepare and cook meals on recipes (minutes)^h^ (range)	20–40	20–50	15–60	15–35	20–40	25–45	35–40	20–23	20–30

^a^Australian states and territories (*n* = 8)—Australian Capital Territory (ACT), New South Wales (NSW), Northern Territory (NT), Queensland (QLD), South Australia (SA), Tasmania (TAS), Victoria (VIC) and Western Australia (WA).

^b^Number of weekly meal options available in May/June 2022.

^c^Based on a meal kit for two people for 3 days/meals, including delivery cost and excluding sign-on discounts.

^d^Based on a meal kit for two people for 3 days/meals and calculated as cost per person per meal (*n* = 6 for the week), includes delivery cost.

^e^Meal types are labels used by the MKSSs, basic definitions include: Family friendly/Kid-approved/Kid-friendly—recipes are generally non-spicy, quick to prepare and include simple ingredients. Low-calorie/Calorie smart—meals that are <599/650 calories per serve. Reduced-carb/Carb smart—meals that contain less carbohydrates per serve. Balanced—contain high-fibre vegetables, wholegrains and lean proteins with minimally processed ingredients. Feed-a-crowd—larger recipes that make double portions of current meal plan by including extra carbohydrates and vegetables. Premium/Gourmet—premium cuts of meat/fish and ingredients, these meals incur additional costs per serve. Ready-to-heat—pre-prepared that require no meal preparation or cooking, just reheating. Nourishing and delicious—high-fibre and fewer refined carbohydrates. Almost ready meals—meal is mostly pre-prepared and requires reheating, some ingredients require minimal prep and cooking.

^f^Data based on three recipes provided during meal kit delivery, other recipes not available online.

^g^Premium/Gourmet/ready-to-heat meals incur additional costs ranging from $0.99 to $5.99 per serving per recipe across the MKSSs.

^h^Excluding Dinnerly™ and Marley Spoon™ monthly main specials which ranged from 1 to 1.5 hours, and You Plate It slow cooker meal reported at 130 minutes from preparation to eat.

### Vegetable content analysis

Recipes from three MKSSs were only accessible through the 1-week subscription and limited to the number of meals delivered (i.e. recipes received with meal kit delivery). Since we could not access a full week of recipes for these MKSSs (Pepper Leaf™, Dinner Sorted™ and My Foodie Box™), these were included in the meal kit description data but excluded from vegetable content analyses. Therefore, of the 231 recipes on offer in a 1-week period across the nine MKSSs (excluding ready-to-heat meals), 179 recipes were included in the analysis.

Table 2 provides an overview of the vegetable content of the recipes included in the analysis for each MKSS (*n* = 6). Both continuous variables (i.e. vegetable serves per person) and categorical variables (i.e. vegetable serve categories) are represented for all recipes (*n* = 179), vegetarian/vegan recipes (*n* = 57) and non-vegetarian/vegan recipes (*n* = 122). Vegetable type and subgroup variety are reported for each MKSS including the proportion of recipes with vegetables from each of the five FoodWorks subgroups.

**Table 2:** Vegetable content of meal kit subscription service (MKSS) recipes (*n* = 179).

	Dinnerly™ (*n* = 50)	Marley Spoon™ (*n* = 45)	HelloFresh™ (*n* = 34)	Every Plate™ (*n* = 22)	Dinner Twist™ (*n* = 16)	You Plate It™ (*n* = 12)	All MKSSs (*n* = 179)
Vegetable serves per person^a^—All recipes (*n* = 179)							
Median (Q1, Q3)	2.0 (1.4, 2.6)	3.2 (2.5, 4.1)	2.8 (1.7, 4.8)	2.8 (1.9, 3.3)	2.2 (1.8, 3.3)	3.0 (1.7, 4.0)	2.5 (1.8, 3.8)
Range (min–max)	0.7–7.0	1.5–6.8	0.7–7.5	1.2–5.4	1.2–5.1	1.2–5.0	0.7–7.5
Proportion of all recipes including vegetable serve categories (*n*, %)							
≤1.0 vegetable serves	7 (14%)	—	5 (15%)	—	—	—	12 (7%)
1.1 to ≤2.0 vegetable serves	20 (40%)	9 (20%)	9 (26%)	6 (27%)	7 (44%)	5 (42%)	56 (31%)
2.1 to ≤3.0 vegetable serves	13 (26%)	8 (18%)	5 (15%)	9 (40%)	3 (19%)	2 (17%)	40 (22%)
3.1 to ≤4.0 vegetable serves	6 (12%)	16 (36%)	5 (15%)	5 (23%)	3 (19%)	2 (17%)	37 (21%)
≥4.1 vegetable serves	4 (8%)	12 (27%)	10 (29%)	2 (9%)	3 (19%)	3 (25%)	34 (19%)
Vegetable serves per person—Vegetarian/vegan recipes (*n* = 57)							
Proportion of vegetarian/vegan recipes (*n*, %)	20 (40%)	15 (33%)	7 (21%)	6 (27%)	6 (38%)	3 (25%)	57 (32%)
Median (Q1, Q3)	2.4 (1.9, 3.5)	3.9 (2.8, 5.5)	5.6 (2.9, 6.1)	3.2 (2.5, 3.6)	2.3 (2, 3.3)	3.8 (1.8, 4.5)	3.1 (2.0, 4.6)
Range (min–max)	0.9– 7.0	1.8–6.8	1.9–7.5	1.4–5.4	2.0–5.1	1.8–4.5	0.9–7.5
Proportion of vegetarian/vegan recipes including vegetable serve categories (*n*, %)							
≤1.0 vegetable serves	2 (10%)	—	—	—	—	—	2 (4%)
1.1 to ≤2.0 vegetable serves	6 (30%)	2 (13%)	1 (14%)	1 (17%)	2 (33%)	1 (33%)	13 (23%)
2.1 to ≤3.0 vegetable serves	6 (30%)	2 (13%)	1 (14%)	2 (33%)	2 (33%)	—	13 (23%)
3.1 to ≤4.0 vegetable serves	3 (15%)	5 (33%)	—	2 (33%)	1 (17%)	1 (33%)	12 (21%)
≥4.1 vegetable serves	3 (15%)	6 (40%)	5 (71%)	1 (17%)	1 (17%)	1 (33%)	17 (30%)
Vegetable serves per person—Non-vegetarian/vegan recipes (*n* = 122)							
Proportion of ­non-vegetarian/vegan recipes (*n*, %)	30 (60%)	30 (67%)	27 (79%)	16 (73%)	10 (63%)	9 (75%)	122 (68%)
Median (Q1, Q3)	1.8 (1.3, 2.2)	3.1 (2.4, 5.7)	2.2 (1.4, 3.8)	2.6 (1.9, 3.1)	2.0 (1.3, 3.2)	3.0 (1.6, 3.4)	2.4 (1.6, 3.3)
Range (min–max)	0.7–4.9	1.5–5.7	0.7–5.6	1.2–4.2	1.2–5.1	1.2–5.0	0.7–5.6
Proportion of non-vegetarian/vegan recipes including vegetable serve categories (*n*, %)							
≤1.0 vegetable serves	5 (17%)	—	5 (19%)	—	—	—	10 (8%)
1.1 to ≤2.0 vegetable serves	14 (47%)	7 (23%)	8 (30%)	5 (31%)	5 (50%)	4 (44%)	43 (35%)
2.1 to ≤3.0 vegetable serves	7 (23%)	6 (20%)	4 (15%)	7 (44%)	1 (10%)	2 (22%)	27 (22%)
3.1 to ≤4.0 vegetable serves	3 (10%)	11 (37%)	5 (19%)	3 (19%)	2 (20%)	1 (11%)	25 (21%)
≥4.1 vegetable serves	1 (3%)	6 (20%)	5 (19%)	1 (6%)	2 (20%)	2 (22%)	17 (14%)
Vegetable type variety—(number of different vegetables provided per recipe)							
Median (range)	2 (1–4)	3 (2–5)	4 (1–6)	3 (2–6)	4 (2–6)	3 (2–5)	3 (1–6)
Vegetable subgroup variety—score out of five based on inclusion of each of the five vegetable subgroups^b^							
Median (range)	2 (1–4)	2 (1–4)	3 (1–5)	3 (2–4)	3 (1–5)	2 (1–3)	2 (1–5)
Proportion of recipes with vegetables from each FoodWorks subgroup^b^ (*n*, %)							
Dark green vegetables	20 (40%)	19 (42%)	24 (71%)	12 (55%)	8 (50%)	7 (58%)	90 (50%)
Red and orange vegetables	35 (70%)	38 (84%)	29 (85%)	20 (91%)	13 (81%)	6 (50%)	141 (79%)
Starchy vegetables	12 (24%)	11 (24%)	16 (47%)	10 (45%)	7 (43%)	5 (42%)	61 (34%)
Legumes	5 (10%)	5 (11%)	4 (12%)	2 (9%)	2 (13%)	0 (0%)	17 (10%)
Other vegetables	35 (70%)	41 (91%)	21 (62%)	17 (77%)	12 (75%)	8 (67%)	134 (75%)

^a^Vegetable serves per person as defined by FoodWorks (informed by Australian Dietary Guidelines modelling system).

^b^Vegetable subgroups as defined by FoodWorks: dark green vegetables—broccoli, bok choy variants, kale, leafy greens, rocket and spinach; red and orange vegetables—capsicum, carrot, pumpkin and tomatoes; starchy vegetables—potato, sweet potato and corn; legumes—black beans, butter beans, cannellini beans, chickpeas, lentils, red kidney beans and white beans; and other vegetables—avocado, beetroot, cabbage, cauliflower, celery, coleslaw, cucumber, eggplant, green beans, leek, lettuce, mushroom, onion, parsnip, peas, radish, snow peas and zucchini.

Across all analysed recipes (*n* = 179), the median number of vegetable serves per person was 2.5, and ranged across services from 2.0 serves for Dinnerly™ up to 3.2 serves for Marley Spoon™ (Table 2). Thirty-two percent (*n* = 57) of all recipes were vegetarian/vegan and provided a median of 3.1 vegetable serves per person across the MKSSs, ranging from 2.3 for Dinner Twist™ to 5.6 for HelloFresh™.

When assessed as a categorical variable, there was notable variability in the proportion of ‘all recipes’ providing high or low vegetable quantities across the different MKSSs. For example, the proportion of all recipes from each MKSS with vegetable serves >3.1 ranged from 63% (Marley Spoon™) to 20% (Dinnerly™). Thirty-eight percent (*n* = 68) of ‘all recipes’ (*n* = 179) included <2.0 standard serves of vegetables per person. Non-vegetarian/vegan meals contributed to a greater proportion of these recipes (43%) and vegetarian/vegan recipes (27%) contributed the least. This ranged across services from 23% (Dinnerly™) of non-vegetarian/vegan recipes with vegetable quantities below 2.0 standard serves to 63% (Marely Spoon™) of non-vegetarian/vegan recipes.

Across the MKSSs, analysed recipes provided a median of 3 different types of vegetables per recipe, ranging from 1 to 6 vegetable types (Table 2). The most frequently provided types of vegetables were mixed leafy greens (including lettuce, kale, rocket, spinach and bok choy variants) (*n* = 89), tomatoes (including raw, diced and passata) (*n* = 76), onion (including shallots) (*n* = 76) and carrots (*n* = 67). Garlic (*n* = 114) was the most frequently provided flavouring, followed by fresh herbs (*n* = 74), tomato paste (*n* = 24) and spring onion (*n* = 22).

A median of two vegetable subgroups were provided across the analysed recipes (*n* = 179) (Table 2). The proportion of recipes providing vegetables from each of the vegetable subgroups varied across the MKSSs. The most frequently included vegetable subgroups across all recipes were ‘red and orange vegetables’ (included in 79% of recipes) and ‘other vegetables’ (included in 75% of recipes). ‘Dark green vegetables’ were included in half (50%) of the recipes. Vegetable subgroups least offered included ‘starchy vegetables’ (34% of recipes) and ‘legumes’ (10% of recipes). Vegetarian/vegan recipes provided three-quarters (71%) of the legume subgroup.

## DISCUSSION

To our knowledge, this is the first study to comprehensively document the Australian MKSS market and provides the first vegetable-specific analysis of MKSS recipes offered over a 1-week period. With inclusion of nine MKSS, our findings highlight the rapid growth and expansion of the Australian MKSS market. Over the past decade, four multi-national and five locally owned MKSSs have emerged in Australia, the most recent established in 2020. The first Australian MKSS paper (published in 2019) (Gibson and Partridge, 2019) described five MKSSs servicing Sydney and Melbourne, of which four were identified in our study, with one having left the market. Our study documents the rapid evolution of MKSSs offerings, with several companies providing a substantial increase in the number and types of weekly meals (i.e. vegan, low-calorie/reduced-carbohydrate and ready-to-heat). For example, HelloFresh™ and Dinnerly™ have increased their weekly recipes from eight, identified in 2019 (Gibson and Partridge, 2019), to over 34 weekly recipes identified in this study. Moreover, our study highlights the diversification and rapidly evolving landscape of MKSSs, with all companies providing an online marketplace for purchasing additional grocery items such as desserts, snacks and drinks.

This study complements and extends previous research (Gibson and Partridge, 2019; Moores *et al.*, 2020) with the addition of analysis of all recipes available in a 1-week period across six MKSSs and by providing a more detailed vegetable analysis. The vegetable content of more than half the MKSSs recipes provided at least half (2.7 serves) of an adult’s daily vegetable requirements (5 serves) (NHMRC, 2013) in one meal and included three different types of vegetables. However, the wide range of vegetable serves across all recipes (ranging from 0.7 to 7.5 serves) documented in our study suggests that a nutritious meal is not guaranteed by ordering MKSSs. A large proportion of recipes (38%) included <2.0 vegetable serves per person. Although all MKSSs offered vegetarian-based meals (32% of all recipes), our findings highlight that almost one-third of these recipes (*n* = 15) included <2.0 serves of vegetables per person. Furthermore, 43% of non-vegetarian/vegan recipes (*n* = 53) also included <2.0 vegetable serves per person. The variability in vegetable content of recipes is an important finding if families are relying on these meals to provide a healthy diet. Australian households typically consume a high proportion of their daily vegetables during the evening meal (Wyse *et al.*, 2011; Nour *et al.*, 2017; Fayet-Moore *et al.*, 2020; Rebuli *et al*., 2020) representing up to three-quarters of adults’ and childrens’ daily vegetable intakes (Rebuli *et al*., 2020).

While the feasibility of implementing health-promoting policies with MKSS companies requires further investigation, it is reasonable to suggest that an opportunity exists for MKSSs to better understand consumer’s constructions of healthy meals and the role vegetables play, and in turn increase vegetable quantity and variety, particularly for non-vegetarian/vegan meals. This would support families to increase their daily vegetable consumption and potentially increase exposure to greater variety of vegetables. In addition, a policy could target the provision of a greater proportion of recipes providing legumes, given that only 10% of recipes incorporated this vegetable subgroup. Legumes are nutritious, relatively cheap, have a long shelf-life and offer a sustainable source of protein. Research suggests increasing familiarity and ability to prepare legumes (Figueira *et al.*, 2019), particularly through practical experience (Lea *et al.*, 2005), may support consumers to overcome commonly perceived barriers to consumption including lack of knowledge and time. MKSSs could educate families by increasing taste exposure, self-efficacy and confidence to incorporate legumes and bean varieties into everyday meals, thus increasing the nutritional quality and environmental sustainability of meals.

Our findings indicate that MKSSs may influence family food provisioning and dietary behaviours through hands-on practical experience. The wide variety of meal types documented in this study may increase meal provider’s confidence and self-efficacy to prepare a range of cuisines and/or foods thus expanding meal repertoires. A systematic review of adult food and cooking interventions reported that a practical skills element was common to interventions reporting long-term behaviour change (Hollywood *et al.*, 2018). Moreover, all MKSSs provided vegetarian recipes which may increase confidence and skills to prepare vegetables, shown to increase the purchasing of a greater variety of household vegetables (Winkler and Turrell, 2010). It is possible that the learning associated with preparing MKSS recipes may, in turn, improve skills to prepare meals, including vegetarian meals, when not using MKSSs, however, this warrants further investigation.

Little is known about how families use MKSSs and modifications that might be made, including vegetable substitutions/additions, when feeding young children (Fraser *et al*., 2021). For example, when children are young and consume small quantities, a family of four (two adults and two young children) may choose to purchase a two-person MKSS meal, in which case vegetable consumption would be lower. Further research could seek to understand how families with young children use MKSSs to explore potential opportunities for MKSSs to promote and increase family vegetable consumption. Given MKSSs are not specifically designed to improve population dietary intakes, families may require support using MKSSs to promote increased family vegetable consumption.

Previous research suggests the cost of MKSSs may limit their reach, disadvantaging those on lower incomes (Gibson and Partridge, 2019; Moores *et al*., 2020; Fraser *et a*l., 2021). The introduction of two lower-price point MKSSs documented in our study may acknowledge cost as a barrier and extend the accessibility of these services to population groups with greater budgetary restraints. In addition, with food waste in Australia costing around AUD$2000–2500 per household/year (Australian Government, 2022), it is possible that the provision of pre-portioned ingredients may assist in reducing household food waste (Heard *et al.*, 2019), and food expenditures overall. Future research could investigate associations between cost and the vegetable or other components of MKSS meals, and whether incorporating legume and bean varieties may be a method for MKSSs to increase the vegetable and protein content of recipes while managing costs.

### Strengths and limitations

A key strength of this study includes the comprehensive analysis of the Australian MKSS market including documentation of all MKSSs, assessment of recipes available during a 1-week period and analysis of vegetable content. The development of a standardized protocol and codebook for data entry ensured vegetable quantities were consistently applied across MKSSs. There are several limitations to be acknowledged. A pragmatic approach informed the study design and therefore limited data collection and analysis to recipes available online or through a meal kit subscription. This resulted in recipes from three MKSSs not being included in the vegetable analyses and therefore were not represented in the data.

Our analysis was limited by the inability to weigh all recipe ingredients with unspecified quantities, therefore informed estimations of vegetable quantities/weights were applied. The focus of this study was to determine the vegetable contribution of MKSS recipes, therefore a full nutritional analysis of MKSS recipes was not conducted. This would be valuable in future as previous Australian research suggests that MKSS recipes are high in sodium and energy (from fat and protein) and low in fibre (Gibson and Partridge, 2019; Moores *et al*., 2020). Finally, our analysis was conducted during May–June 2022 (autumn–winter in Australia) and does not account for seasonal differences in vegetable availability and cost.

## CONCLUSION

This study provides a contemporary overview of the rapidly evolving MKSS market in Australia and highlights the potential for harnessing MKSSs to influence family vegetable consumption. From a nutrition perspective, our findings highlight that MKSSs may support families in meeting daily vegetable recommendations, particularly if families select recipes with a greater number and variety of vegetables. However, an opportunity exists for researchers to work with MKSSs to strengthen their opportunity to market healthy meals and to improve the vegetable quantity and variety of meals. Combined with the many opportunities known to influence vegetable consumption, such as enabling home cooking and facilitating food literacy development, MKSSs may be a viable mechanism to support family mealtime vegetable consumption. However, there is a need for further research to explore how families, especially those with young children, use meal kits, to better understand the potential of MKSSs to improve family nutrition.

## Data Availability

The data that support the findings of this study are available from the corresponding author upon reasonable request.
